# Poly(3-hydroxybutyrate-co-3-hydroxyvalerate) co-produced with l-isoleucine in *Corynebacterium glutamicum* WM001

**DOI:** 10.1186/s12934-018-0942-7

**Published:** 2018-06-15

**Authors:** Wenjian Ma, Jianli Wang, Ye Li, Lianghong Yin, Xiaoyuan Wang

**Affiliations:** 1 State Key Laboratory of Food Science and Technology, Jiangnan University, 1800 Lihu Avenue, Wuxi, 214122 China; 2 International Joint Laboratory on Food Safety, Jiangnan University, Wuxi, 214122 China; 3 Key Laboratory of Industrial Biotechnology, Ministry of Education, School of Biotechnology, Jiangnan University, Wuxi, 214122 China

**Keywords:** *Corynebacterium glutamicum*, l-Isoleucine, PHBV, 3HV fraction, Propionyl-CoA

## Abstract

**Background:**

Co-production of polyhydroxyalkanoate (PHA) and amino acids makes bacteria effective microbial cell factories by secreting amino acids outside while accumulating PHA granules inside. Poly(3-hydroxybutyrate-co-3-hydroxyvalerate) (PHBV) is one of the PHAs with biocompatibility and fine mechanical properties, but its production is limited by the low level of intracellular propionyl-CoA.

**Results:**

l-Isoleucine producing *Corynebacterium glutamicum* strain WM001 were analyzed by genome and transcriptome sequencing. The results showed that the most over-expressed genes in WM001 are relevant not only to l-isoleucine production but also to propionyl-CoA accumulation. Compared to the wild-type *C. glutamicum* ATCC13869, the transcriptional levels of the genes *prpC2, prpD2,* and *prpB2*, which are key genes relevant to propionyl-CoA accumulation, increased 2^6.7^, 2^5.8^, and 2^8.4^-folds in WM001, respectively; and the intracellular level of propionyl-CoA increased 16.9-fold in WM001. When the gene cluster *phaCAB* for PHA biosynthesis was introduced into WM001, the recombinant strain WM001/pDXW-8-*phaCAB* produced 15.0 g/L PHBV with high percentage of 3-hydroxyvalerate as well as 29.8 g/L l-isoleucine after fed-batch fermentation. The maximum 3-hydroxyvalerate fraction in PHBV produced by WM001/pDXW-8-*phaCAB* using glucose as the sole carbon source could reach 72.5%, which is the highest reported so far.

**Conclusions:**

Genome and transcriptome analysis showed that *C. glutamicum* WM001 has potential to accumulate l-isoleucine and propionyl-CoA pool. This was experimentally confirmed by introducing the *phaCAB* gene cluster into WM001. The recombinant strain WM001/pDXW-8-*phaCAB* produced high levels of PHBV with high 3-hydroxyvalerate fraction as well as l-isoleucine. Because of its high level of intracellular propionyl-CoA pool, WM001 might be used for producing other propionyl-CoA derivatives.

**Electronic supplementary material:**

The online version of this article (10.1186/s12934-018-0942-7) contains supplementary material, which is available to authorized users.

## Background

l-Isoleucine is used as a component of cosmetics, animal feed additives, and pharmaceuticals [1]. Industrial producers of l-isoleucine were mostly *Corynebacterium glutamicum* [2]. In *C. glutamicum*, l-isoleucine synthesis from l-aspartate involves ten reactions, five of them are feedback-inhibited by l-threonine or l-isoleucine [2]. Several l-isoleucine producers have been obtained by random mutation, site-directed mutagenesis, and rational metabolic engineering [1, 3–10]. Polyhydroxyalkanoates (PHAs) are natural biodegradable and biocompatible polyesters with piezoelectricity and flexible mechanical properties and are potential alternatives for petroleum-based plastics [11]. PHAs accumulated in various microorganisms and plants for storing carbon and energy under nutrient imbalance [12] and could be naturally degraded within 100 h in the soil [11].

The major PHA is poly(3-hydroxybutyrate) (PHB) [13], however, the later discovered poly(3-hydroxybutyrate-co-3-hydroxyvalerate) (PHBV) owns better biocompatibility and mechanical properties, which makes PHBV more proper for the pharmaceutical application [14]. 3-Hydroxyvalerate (3HV) derived from propionyl-CoA is a crucial intermediate of PHBV; high fraction of 3HV in PHBV improves strength and flexibility of the polyester [15]. However, 3HV fractions are usually low in most PHBV products due to the propionyl-CoA limitation (Table 1). Improving the 3HV fraction in PHBV could be achieved by addition of the related precursors of propionyl-CoA, such as propionate [16], valerate [17], succinate [18], or l-threonine [19]; but such addition increases the cost and retards the cell growth (Table 1). Study of PHBV life cycle indicates that biopolymers like PHBV could be largely independent of fossil fuels in the future [20]. Current production scale of PHBV is about 2000 tons per year but its expectable production scale is around 50,000 tons per year.

**Table 1:** Comparison of the reported PHBV producers

Strain	Carbon source	3HV fraction (mol/mol) (%)	DCW (g/L)	References
*Agrobacterium *sp*. *	Propionate	50	11	[59]
*Salmonella typhimurium*	Glycerol, propionate	30.6	–	[54]
*Alcaligenes* sp.	Glucose, levulinic acid	76.5	4.03	[60]
*R. eutropha*	Glucose, valerate	62.7	110.2	[17]
*R. aetherivorans*	Acetate	79	2.5	[47]
*H. mediterranei*	Starch	13.4	7.01	[61]
*C. glutamicum*	Glucose, propionate	28	12.0	[22]
*E. coli*	Glucose	25.4	–	[51]
*R. eutropha*	Glucose	26.0	132.8	[62]
*Bacillus* spp.	Glucose, l-threonine	48	2.02	[19]
*C. necator*	Glucose, levulinic acid, sodium propionate	80	–	[56]
*E. coli*	Glucose, propionate	67.9	2.9	[16]
*C. glutamicum*	Glucose	75.2	76	This work

Since Gram-negative bacteria synthesize large amount of immunogenic lipopolysaccharide which might contaminate the products and become a latent hazard in medical and food container application [21], PHBV are usually produced by using Gram-positive bacteria, such as *Nocardia*, *Rhodococcus*, *Bacillus*, *Corynebacterium*. *Nocardia* and *Rhodococcus* could naturally accumulate PHBV but are commercially non-viable [20], *Bacillus* could produce PHBV [19] but requires addition of extra threonine and cyanocobalamin, *Corynebacterium* could produce PHBV [22] but requires the addition of propionate in the medium. PHA could be co-produced with amino acids, such as l-glutamate [23], l-tryptophan [24], l-arginine [25], and succinate [26]. The co-productions positively affected the transcription of key enzymes, increased the product yield, rearranged the cofactor flux, and improved the cell growth.

In this study, the gene cluster *phaCAB* which contains the three key genes for PHA biosynthesis into a l-isoleucine producing *C. glutamicum* strain WM001, resulting in the strain WM001/pDXW-8-*phaCAB.* WM001/pDXW-8-*phaCAB* produced high levels of PHBV with high 3HV fraction as well as l-isoleucine, using glucose as the sole carbon source.

## Methods

### Strains, plasmids, and genetic methods

Bacterial strains used in this study are listed in Table 2. l-isoleucine-producing *C. glutamicum* strain WM001 (CCTCC No. M2016303) was originally isolated from soil, and is closely related to *C. glutamicum* strain ATCC13869, based on their 16S rDNA sequences. Plasmid preparation kit, gel extraction kit, and DNA purification kit were purchased from Sangon Biotech (Shanghai, China). *phaCAB* cluster was amplified from pBHR68 using primers *phaCAB*-F (5′-CTGGAATTCAGAAGGAGAATCAAATCATGGCGACCGG-3′ (restriction site underlined)) and *phaCAB*-R (5′-CCGCTCGAGAGGTCAGCCCATATGCAGG-3′ (restriction site underlined)). The PCR product was digested with *Eco*RI and *Xho*I and ligated into pDXW-8 [27] which was similarly digested, resulting in pDXW-8-*phaCAB.* Similarly, the *phaAB* cluster was amplified using primer pairs *phaAB*-F (5′-CGGAATTCAGAAGGAGATATACCATGACTGACGTTGTCATCG-3′ (restriction site underlined)) and *phaAB*-R (5′-CCGCTCGAGAGGTCAGCCCATATGCAGG-3′ (restriction site underlined)) and ligated into pDXW-8, resulting in pDXW-8-*phaAB*, the gene *phaA* was amplified using primer pairs *phaA*-F (5′-CGGAATTCAGAAGGAGATATACCATGACTGACGTTGTCATCG-3′ (restriction site underlined)) and *phaA*-R (5′-CTGCTCGAGACCCCTTCCTTATTTGCGC-3′ (restriction site underlined)) and ligated into pDXW-8, resulting in pDXW-8-*phaA*. Plasmids pDXW-8-*phaA,* pDXW-8-*phaAB,* and pDXW-8-*phaCAB* were then transformed to *C. glutamicum* ATCC13869 and *C. glutamicum* WM001. *E. coli* DH5α strains were grown at 37 °C in Luria–Bertani medium (10 g/L tryptone, 5 g/L yeast extract, and 10 g/L NaCl, pH 7.2). *C. glutamicum* strains were grown at 30 °C in LBHIS medium (10 g/L NaCl, 10 g/L peptone, 5 g/L yeast extract, 18.5 g/L brain heart infusion, and 91 g/L d-sorbitol). When necessary, 30 μg/mL kanamycin was added to the medium to maintain the plasmids, and 0.5 mM isopropyl β-d-thiogalactoside was added to the medium for induction.

**Table 2:** Strains and plasmids used in the study

Strains or plasmids	Description	Sources
Strains		
DH5α	*E. coli*	NEB
DH5α/pDXW-8	DH5α harboring pDXW-8	This study
DH5α/pDXW-8-*phaA*	DH5α harboring pDXW-8-*phaA*	This study
DH5α/pDXW-8-*phaAB*	DH5α harboring pDXW-8-*phaAB*	This study
DH5α/pDXW-8-*phaCAB*	DH5α harboring pDXW-8-*phaCAB*	This study
ATCC13869	Wild-type *C. glutamicum*	ATCC
ATCC13869/pDXW-8	ATCC13869 harboring pDXW-8	This study
ATCC13869/pDXW-8-*phaCAB*	ATCC13869 harboring pDXW-8-*phaCAB*	This study
WM001	*C. glutamicum* l-isoleucine producer	CCTCC
WM001/pDXW-8	WM001 harboring pDXW-8	This study
WM001/pDXW-8-*phaA*	WM001 harboring pDXW-8-*phaA*	This study
WM001/pDXW-8-*phaAB*	WM001 harboring pDXW-8-*phaAB*	This study
WM001/pDXW-8-*phaCAB*	WM001 harboring pDXW-8-*phaCAB*	This study
Plasmids		
pBHR68	Template plasmid of *phaCAB* cluster	[63]
pDXW-8	Shuttle vector between *E. coli* and *C. glutamicum*	[27]
pDXW-8-*phaA*	pDXW-8 harboring *phaA*	This study
pDXW-8-*phaAB*	pDXW-8 harboring *phaAB*	This study
pDXW-8-*phaCAB*	pDXW-8 harboring *phaCAB*	This study

### Batch fermentation

*Corynebacterium glutamicum* cells were inoculated at 30 °C for 36 h on the agar plate containing 5 g/L glucose, 10 g/L tryptone, 5 g/L beef extract, 5 g/L yeast extract, and 5 g/L NaCl. A single colony was inoculated in 25 mL seed medium in a 500-mL flask for 18 h until OD_562_ reached 10. The seed culture was then inoculated into 25 mL fermentation medium in 500-mL flasks, the initial OD_562_ was adjusted to 1. Seed medium contains 30 g/L glucose, 5 g/L (NH_4_)_2_SO_4_, 1 g/L KH_2_PO_4_, 0.5 g/L MgSO_4_, and 30 g/L corn steep liquor, pH 7.2 adjusted with 5 M NaOH. Flask fermentation medium contains 130 g/L glucose, 35 g/L (NH_4_)_2_SO_4_, 1 g/L KH_2_PO_4_, 0.5 g/L MgSO_4_ and 15 g/L corn steep liquor, initial pH 7.2 adjusted with 5 M NaOH and maintained with 20 g/L CaCO_3_. Tryptone and yeast extract were purchased from Oxoid (Basingstoke, UK), and corn steep liquor from North China pharmaceutical corporation (Shijiazhuang, China). Other reagents were purchased from Sinopharm chemical reagent corporation. 30 µg/mL kanamycin was added in both seed and fermentation medium before inoculation, and 0.5 mM IPTG was added 6 h after inoculation. Flask cultivation was performed in a rotary shaker at 200 rpm and 30 °C for 96 h. Samples were collected every 12 h to determine the optical density and levels of glucose, organic acids, amino acids and PHA.

### Fed-batch fermentation

*Corynebacterium glutamicum* cells were first cultivated in 60 mL seed medium in 500-mL flasks for 18 h, then transferred into a 3-L fermentor (New Brunswick Scientific, New Brunswick, New Jersey) with 1.14 L fermentation medium. The fed-batch fermentation medium contains 130 g/L glucose, 10 g/L (NH_4_)_2_SO_4_, 1 g/L KH_2_PO_4_, 0.5 g/L MgSO_4_ and 15 g/L corn steep liquor; initial pH was adjusted to 7.2 with 5 M NaOH and maintained with 50% ammonia. After 6 h, 0.5 mM IPTG was added for induction. Samples were collected every 12 h to determine the optical density and levels of glucose, organic acids, amino acids and PHA. The aeration rate was 1 L/min. The dissolved oxygen was cascaded to the speed of revolution (400 to 800 rpm) and controlled as 30% for cell growth before 24 h, then 15% for PHA and l-isoleucine production. The residual glucose was maintained above 40 with 500 g/L glucose solution. During the fermentation, too much foam could cause serious culture spill. This often happened during log phase because of the rapid cell growth. 3–4 drops of anti-foam solution were added several times to inhibit foam growing, but too much anti-foam (more than 15 drops) would be harmful to cell growth. If the foam keeps growing, the aeration rate can be decreased to 0.5 L/min or under.

### Determination of amino acids, glucose, and organic acids

For amino acids determination, reverse phase high-pressure liquid chromatography (HPLC) was employed with an Agilent 1200 system (Agilent Technologies, Waldbronn, Germany) equipped with Thermo Hypersil ODS-2 column (5 μm particle, 250 mm × 4.6 mm) (Cheshire, UK) and diode array detection system. Buffer A contains 955 mL 0.1 M sodium acetate, 5 mL tetrahydrofuran and 0.2 mL triethylamine per liter, pH 7.2. Buffer B contains 200 mL 0.1 M sodium acetate (pH 7.2), 400 mL methanol and 400 mL acetonitrile per liter. Sample broths were centrifuged at 10,000 rpm for 5 min, the supernatants were diluted 20 times with trichloroacetic acid and went through 0.22 µm membrane filters. The supernatants were subsequently derivatized with *o*-phthalaldehyde reagent solution (Agilent Technologies, Waldbronn, Germany) and then detected at 338 nm with a flow rate of 1 mL/min.

For glucose determination, SBA-40C immobilized enzyme biosensor (Shandong province academy of sciences, China) was employed. Supernatants were diluted 100 times with distilled water, and 25 µL was injected.

For organic acids determination, Agilent 1260 HPLC was employed for intracellular and extracellular organic acids quantification equipped with Diamonsil C18 column (5 μm, 250 mm × 4.6 mm No. 99603) (DiKMA technology, Beijing, China). A linear gradient elution procedure was employed as methanol:H_2_O:phosphate (from 5:95:0.05 to 60:40:0.05) in 20 min. Samples were detected with an ultraviolet detector at emission wavelengths 210 nm with a flow rate of 0.9 mL/min.

### Qualitative and quantitative analysis of PHBV

For PHA granules observation, samples were treated according to transmission electron microscopy (TEM) specimen preparation protocol [28], and the specimen was imaged with a JEOL JEM 2100 (JEOL Ltd., Tokyo, Japan).

For identification and quantification of PHBV, GC-2010 plus system (Shimadzu, Japan) was employed with a DB-WAX column (30 m × 0.32 mm) (Agilent Technologies, Waldbronn, Germany) and a flame ionization detector, and the injection temperature was 250 °C. Cells were harvested by centrifugation at 10,000 rpm for 5 min, washed twice with pH 7.2 phosphate-buffered saline then lyophilized for 48 h. About 20 mg lyophilized cells, 2 mL methanol (with 3% H_2_SO_4_) and 2 mL chloroform were added to esterification tubes and treated in boiled water for 6 h. 1 mL distilled water was added to esterification tubes at room temperature, and rotary vibrated for 5 min, then 0.5 mL of organic phase was collected and filtrated with 0.22 µm filters (Sartorious, Germany). Calibration curves were constructed with commercially available PHB and PHBV (Sigma-Aldrich, Saint Louis, Missouri).

### Transcriptome analysis

*Corynebacterium glutamicum* ATCC13869 and WM001 were cultured in a 3-L bioreactor, and cells were harvested at late log phase by centrifugation at 10,000 rpm for 5 min, washed twice with PBS pH 7.2, then resuspended in RNA safer stabilizer reagent from Wegene (Shanghai, China). Illumina TruSeq Ribo-Zero™ Stranded Total RNA Sample Preparation kit was used for cDNA library preparation, and biotinylated oligos combined with Ribo-Zero™ rRNA removal beads were used to remove ribosome RNA. The RNA was fragmented into small pieces using divalent cations. Taking these short fragments as templates, random hexamer primer were used to synthesize the first-strand cDNA. The second strand cDNA was synthesized with DNA Polymerase I and RNase H. Short fragments were purified with QiaQuick PCR extraction kit and resolved with elution buffer for end reparation and adding poly(A). After that, the short fragments were connected with sequencing adapters. For amplification with PCR, we selected suitable fragments as templates, with respect to the result of agarose gel electrophoresis. At last, the library was sequenced with Illumina HiSeq™ 2000. The complete genome of *C. glutamicum* ATCC 13032 was used as the reference genome for RNA-sequence reads sequence alignment. The expression level of each gene was calculated using the reads per kilobases per million reads, the analysis results with a *p* value < 0.05 were corrected by the false discovery rate, which was set to < 0.001. RNA-seq transcriptome analysis was conducted by BGI genomics (Shenzhen, China).

### Determination of acetyl-CoA and propionyl-CoA

The sample preparation procedures and LC/MS methods were described in the previous studies [29–31] with modifications. *C. glutamicum* cells were cultured in a 500-mL flask for 24 h then transferred to pre-chilled Eppendorf tubes and centrifuged at 10,000 rpm and 0 °C for 30 s, the sediments were quickly washed twice with cold 0.9% NaCl solution, and resuspended in 1 mL mixture of methanol, acetonitrile and ddH_2_O (45:45:10, v/v/v) at − 20 °C with 0.1 M formic acid for quenching, and then ultrasonicated at 0 °C for 5 min (60 cycles of 2 s running and 3 s interval). The supernatants were filtrated with 0.22 µm filter and directly injected into a TSQ Quantum Ultra (Thermo Scientific, San Jose, CA, USA) with commercially available acetyl-CoA and propionyl-CoA (Sigma-Aldrich, Saint Louis, Missouri) as standards. Buffer A was 5 mM ammonium acetate (Sigma-Aldrich, Saint Louis, Missouri). Buffer B was methanol (Sigma-Aldrich, Saint Louis, Missouri). Samples were eluted at a flow rate of 0.4 mL/min, acetyl-CoA (parent ion m/z = 810, product ion m/z = 303) and propionyl-CoA (parent ion m/z = 824, product ion m/z = 317) were detected and quantified in multi-reaction monitoring (MRM) mode according to the calibration curve. The data were analyzed and exhibited with Xcalibur.

## Results and discussion

### Genome sequencing and transcriptome analysis of *C. glutamicum* WM001

*Corynebacterium glutamicum* has been remarkable platform bacteria for producing l-isoleucine [32]. l-Isoleucine-producing *C. glutamicum* strain WM001 was originally isolated from soil, and is closely related to *C. glutamicum* strain ATCC13869, based on their 16S rDNA sequences. In this study, the genome of WM001 was sequenced with single-molecule real-time sequencing and deposited to the NCBI (NZ_CP022394.1, https://www.ncbi.nlm.nih.gov/nuccore/CP022394). Profiles of the strain are available in NCBI with Biosample No. SAMN07337837 and Bioproject Accession No. PRJNA393604. Sequencing data were assembled and analyzed with Prodigal [33], HGAP [34], tRNAscan-SE [35], and Infernal 1.1 [36]. As a result, a single circular chromosome of 3,319,925 bp was generated with 54.0% GC content and no plasmids. The GC content of *C. glutamicum* WM001 is similar to the GC content of *C. glutamicum* ATCC13032 (53.8%), ATCC14067(54.1%), and ATCC13869 (54.2%) [37]. There were 3070 protein-coding genes, 18 tRNAs, 60 rRNAs and 18 other non-coding RNAs in the genome of WM001.

Proteome analysis between WM001 and ATCC13869 has been studied but the mechanism of WM001 producing excessive l-isoleucine remains unclear [38]. To find out the global metabolic differences between WM001 and ATCC13869, transcriptome analysis was performed. Samples of WM001 and ATCC13869 were harvested at 24 h (late exponential stage) when the dry cell weights reached 33 and 36 g/L, respectively. Regulation threshold was set as twofold, as a result, 406 genes were up-regulated and 237 genes were down-regulated in WM001.

**Fig. 1:**
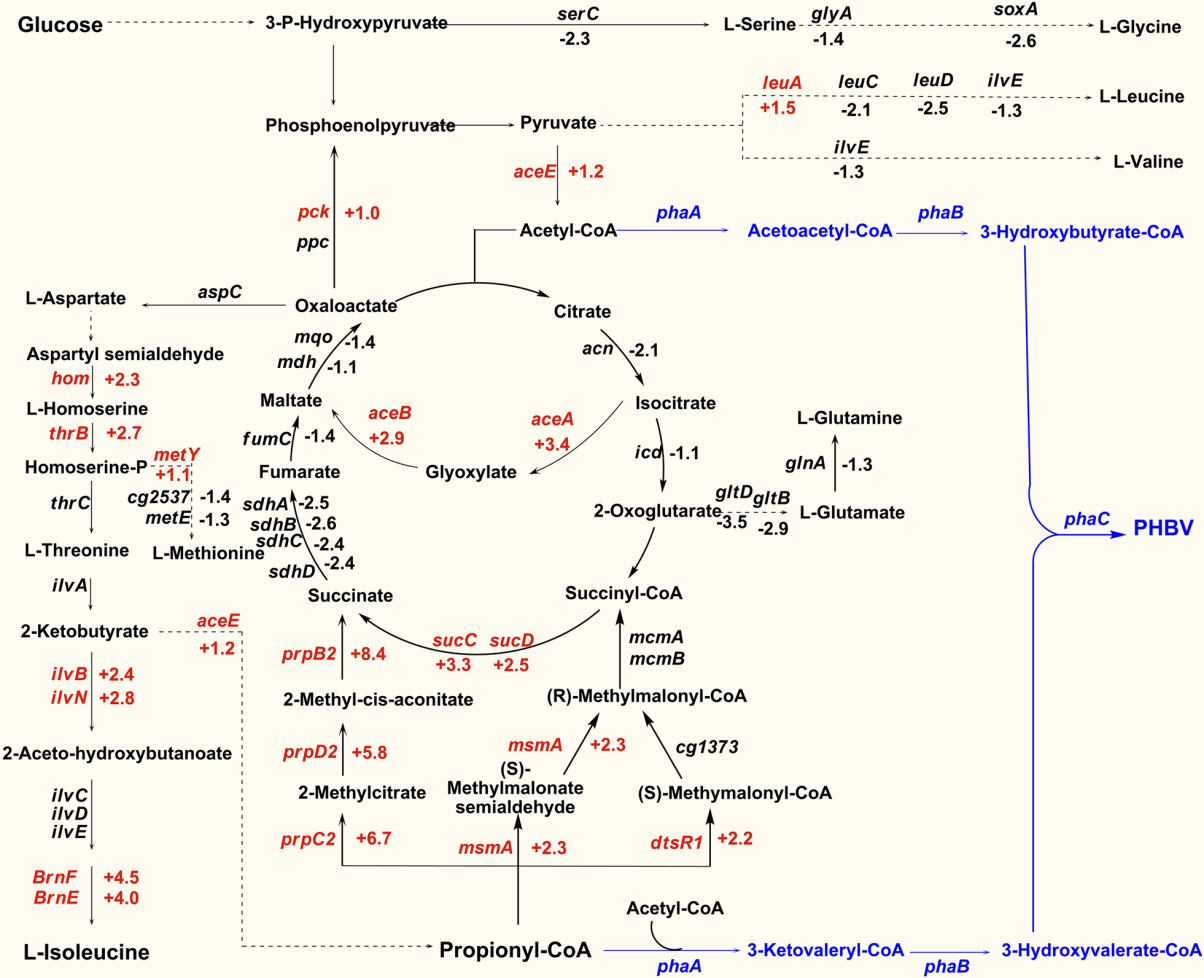
The biosynthetic pathways of l-isoleucine and metabolism of propionyl-CoA in *C. glutamicum.* The genes that were significantly regulated in *C. glutamicum* WM001 are shown together with their log_2_R values, using *C. glutamicum* ATCC13869 as a control. The up-regulated genes are shown in red, while the down-regulated genes are shown in black. The reactions shown in blue exist in WM001/pDXW-8-*phaCAB* but not in *C. glutamicum* WM001

Transcriptome analysis results were shown in Fig. 1. As expected, *ilvN*, *ilvB*, *thrB*, *hom*, which related to l-isoleucine synthesis [10] were up-regulated by 2^2.8^, 2^2.4^, 2^2.7^, and 2^2.3^-folds, respectively. l-Isoleucine exporter encoded by *brnFE* [8, 39] were significantly up-regulated 2^4.5^-fold (*brnF*) and 2^4.0^-fold (*brnE*), which enhanced the secretion of l-isoleucine. This explains the high production of l-isoleucine in *C. glutamicum* strain WM001, and also suggests that BrnFE play an important role on l-isoleucine production in *C. glutamicum* [40]. In addition, the down-regulated of *metE, leuC, leuD, ilvE, serC, glnA, gltB, gltD* reduced the metabolic flux towards other by-products [41, 42], thereby revealing the mechanism that extracellular levels of related amino acids in WM001 were much lower than that in wild-type [38]. Thus, the blocked and reserved carbon flux could be channeled towards l-isoleucine. On the other hand, most TCA cycle genes like *acn, icd, sdhA,sdhB, sdhC, sdhD, fumC, mqo, mdh* were down-regulated 2^2.1^, 2^1.1^, 2^2.5^, 2^2.6^, 2^2.4^, 2^2.4^, 2^1.4^, 2^1.4^, and 2^1.1^-folds, respectively whereas *sucC* and *sucD* were up-regulated 2^3.3^ and 2^2.5^-folds due to catabolism of l-isoleucine and other amino acids. As the down-regulated TCA cycle genes contributed to the l-lysine production in *C. glutamicum* [43], they might also affect l-isoleucine production. In addition, the changes on the regulation of oxidative phosphorylation, glycolysis pathway, pyruvate metabolism, amino acids biosynthesis from transcriptome profiling provide the integrated information of WM001 metabolism (Additional file 1: Table S2).

Interestingly, genes *prpB2, prpC2, prpD2, dtsR1,* and *msmA* encoding the enzymes for propionyl-CoA catabolism were up-regulated 2^8.4^, 2^6.7^, 2^5.8^, 2^2.2^, and 2^2.3^-folds, respectively (Fig. 1). This significantly activated propionyl-CoA catabolism suggests there might be excessive propionyl-CoA, i.e. a huge propionyl-CoA pool, in *C. glutamicum* WM001 cells.

### Overexpressing the *phaCAB* cluster not only produced PHA but also improved l-isoleucine production

In previous studies, PHB accumulation increased 23% l-glutamate [23], 21% succinate [26], 12% l-tryptophan [24], and 21% l-arginine yield [25], respectively. To test whether PHA accumulation could enhance l-isoleucine production as in these reports, and whether WM001 could use the propionyl-CoA pool to produce PHBV rather than PHB, the *phaCAB* cluster was expressed in WM001. After 96 h, l-isoleucine production and glucose consumption of the recombinants were analyzed (Fig. 3). WM001/pDXW-8-*phaCAB* produced 9.58 g/L l-isoleucine while the control only reached 6.65 g/L (Fig. 3a) at 96 h. Glucose consumption in WM001/pDXW-8 and WM001/pDXW-8-*phaCAB* followed the same pattern (Fig. 3b) but less glucose was consumed in WM001/pDXW-8-*phaCAB*. The l-isoleucine yield on glucose in WM001/pDXW-8-*phaCAB* increased 65% compared to the control. Since up-regulation of l-tryptophan and l-arginine synthesis genes were observed when *phaCAB* expressed in *E. coli* and *C. crenatum* [24, 25], transcriptional levels of l-isoleucine biosynthesis genes were determined by RT-PCR. The results showed that *lysC*, *hom*, *ilvA,* and *ilvBN* genes were up-regulated 2^1.7^, 2^1.4^, 2^1.5^, and 2^1.1^-folds in WM001/pDXW-8-*phaCAB*, respectively. Our results were consistent with the above-mentioned studies, suggesting *phaCAB* expression could lead to up-regulation of l-isoleucine biosynthesis genes in *C. glutamicum*.

**Fig. 2:**
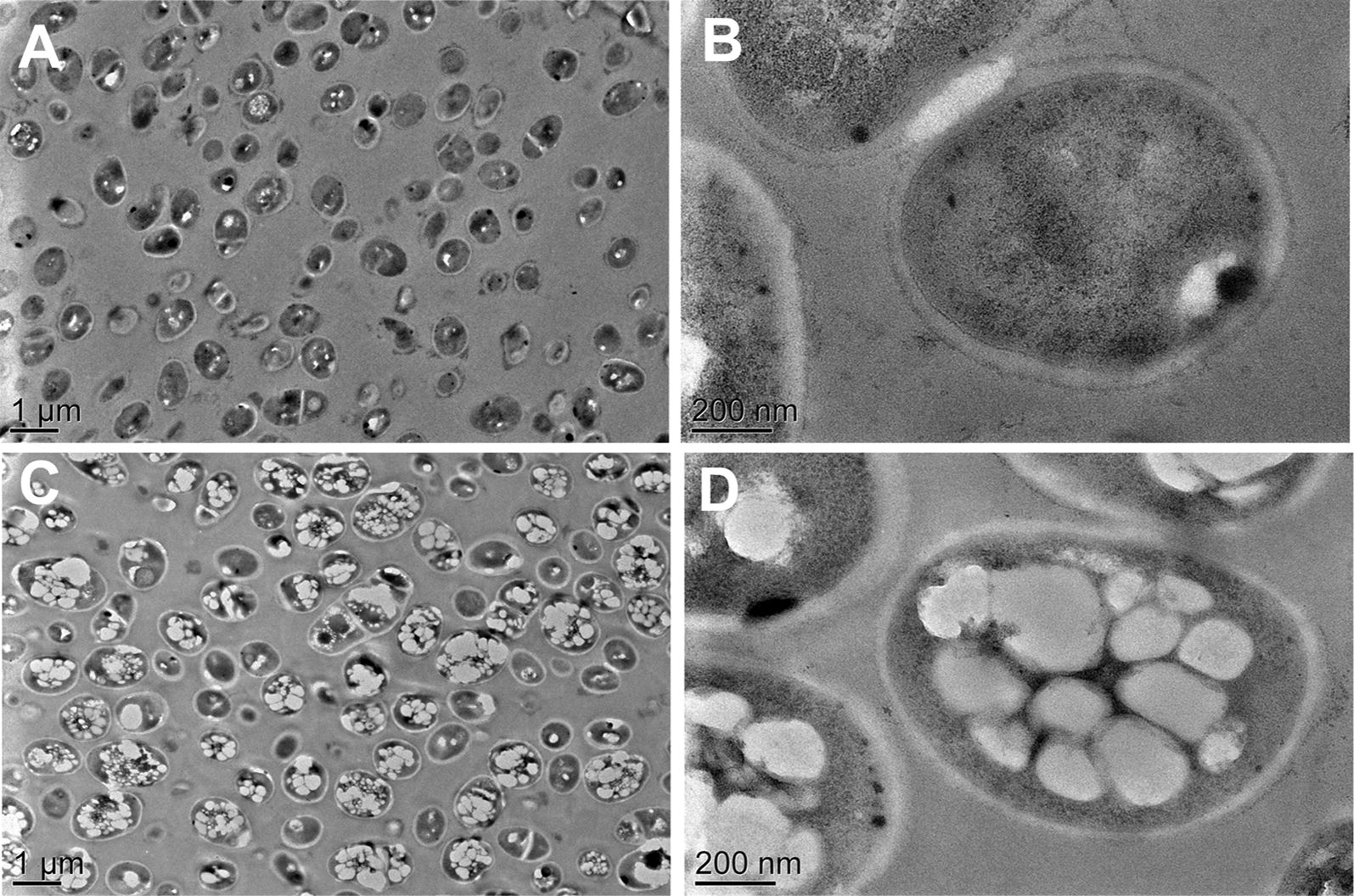
TEM analysis of *C. glutamicum* WM001/pDXW-8 and WM001/pDXW-8-*phaCAB* cells. Intracellular PHA granules were observed in WM001/pDXW-8-*phaCAB* but not in WM001/pDXW-8. **A** WM001/pDXW-8; **B** WM001/pDXW-8; **C** WM001/pDXW-8-*phaCAB*; **D** WM001/pDXW-8-*phaCAB*

**Fig. 3:**
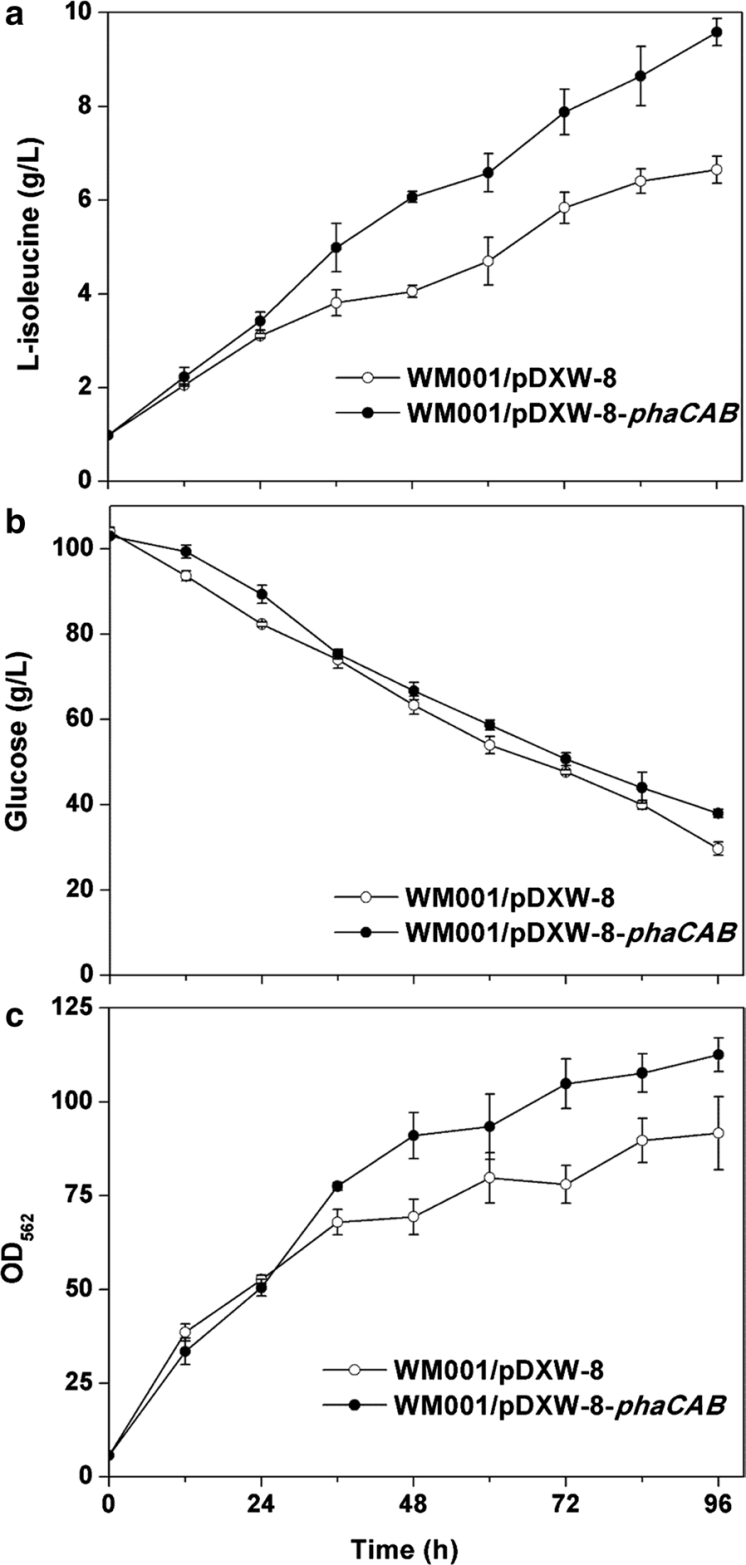
Batch fermentation profiles of WM001/pDXW-8 and WM001/pDXW-8-*phaCAB* cells. **a** The titer of l-isoleucine; **b** glucose; **c** OD_562_

PHA production in WM001/pDXW-8-*phaCAB* was confirmed by TEM analysis (Fig. 2) and SEM analysis (Additional file 1: Figure S1). PHA granules were observed in WM001/pDXW-8-*phaCAB* but not in WM001/pDXW-8. These PHA granules occupied about 70% of the cell volume, and the cells containing PHA are larger than those containing no PHA (Fig. 2 and Additional file 1: Figure S1). During fermentation, WM001/pDXW-8-*phaCAB* cells grew better than the control (Fig. 3c). PHA accumulation was found to facilitate the growth of *E. coli* [26], *Pseudomonas oleovorans* [44], and *Aeromonas hydrophila* [45], possibly because PHA synthesis improves resistance against stress through globe regulators, such as RpoS [44]. Although high levels of l-isoleucine production significantly inhibited the cell growth in our previous study [46], PHA synthesis can recover the cell growth and enhance l-isoleucine production simultaneously in *C. glutamicum*.

The gene cluster *phaCAB* consists of three genes *phaC*, *phaA,* and *phaB*. In this study, to investigate which gene or combinations lead to the enhancement of l-isoleucine, WM001/pDXW-8-*phaA* and WM001/pDXW-8-*phaAB* which strengthened the supply of acetoacetyl-CoA and 3-hydroxybutyrate-CoA were similarly constructed respectively. After checking *phaA* and *phaAB* were well expressed via SDS-PAGE (Additional file 1: Figure S2). We tested both recombinants with batch fermentation as we previously tested WM001/pDXW-8-*phaCAB*. After 72 h fermentation, WM001/pDXW-8-*phaA* and WM001/pDXW-8-*phaAB* showed no significant enhancement of l-isoleucine (Additional file 1: Table S1), that indicates it was the synthesis of PHA polymers rather than any intermediates that improved l-isoleucine production in WM001.

### WM001/pDXW-8-*phaCAB* produces PHBV with a high 3HV fraction

The 3HV fractions in bio-based PHBV polymers were quite low without adding related precursors. Therefore several studies were conducted to improve 3HV fraction (Table 1). Since propionyl-CoA is the most crucial substrate of 3HV, the propionyl-CoA pool size is very important. Unfortunately, endogenous propionyl-CoA is usually limited for its rapid conversion to succinate and succinyl-CoA (Fig. 1). Adding propionyl-CoA precursors like propionate, levulinic acid, threonine, succinate, valerate is the direct way to enhance 3HV fraction but the additions not only raise the cost but also inhibit the cell growth because of the toxicity of the precursors [22]. Without adding propionyl-CoA precursors, some microorganisms could produce PHBV from glucose, toluene, starch, acetate, but these microorganisms require extra supplements such as mevinolin and sodium chloride, which also brings extra costs and difficulties [47–50]. Some engineered microorganisms could use endogenous propionyl-CoA through the combination of citramalate pathway and threonine biosynthesis pathway but the 3HV fraction only reached 25.4% or less [51]. In Gram-positive bacteria, PHBV production without adding related precursor is not reported yet.

**Fig. 4:**
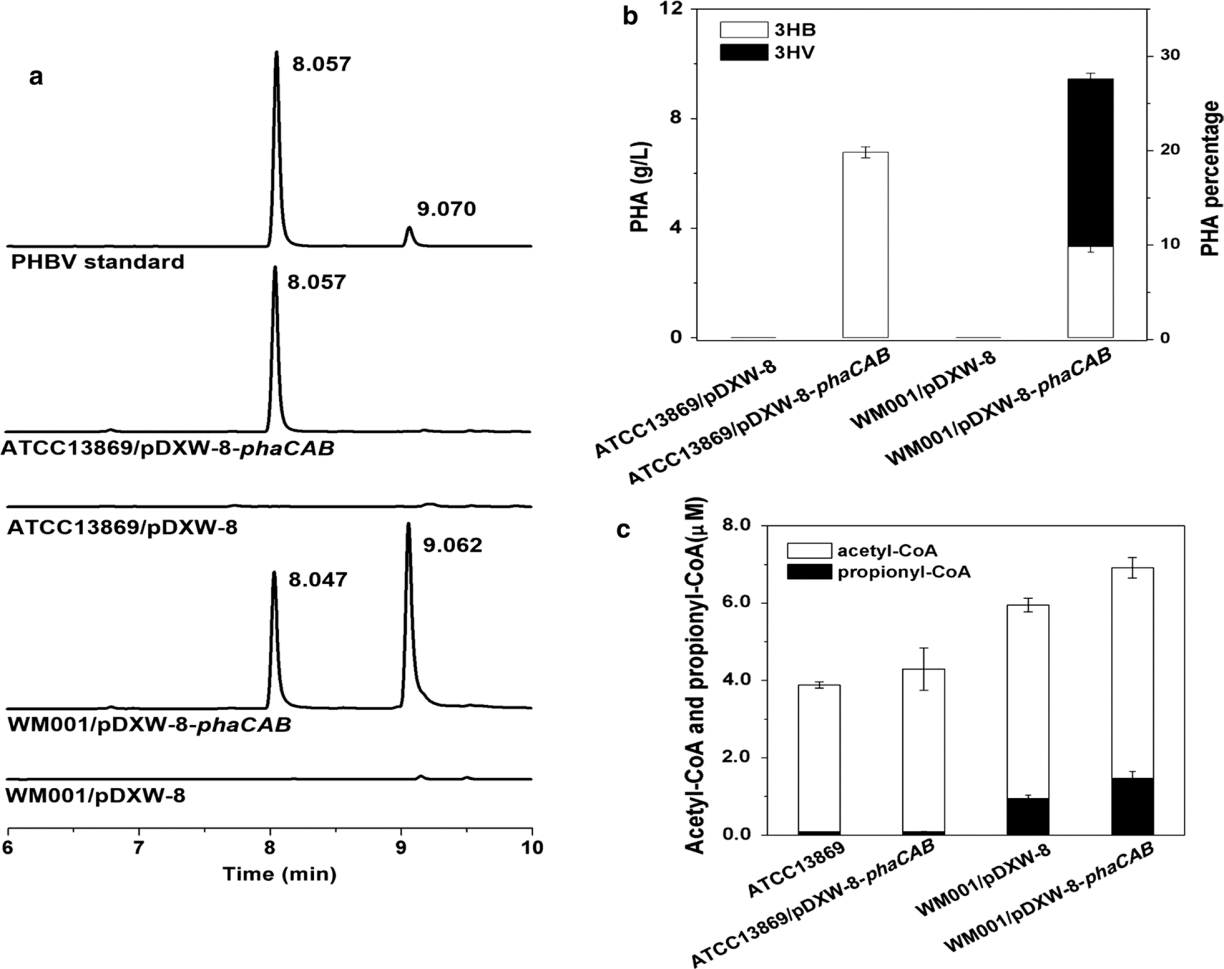
**a** Gas chromatography analysis of PHAs produced by ATCC13869 and WM001 strains under same culture condition after 96 h of batch culture. From top to bottom: PHBV standard (3HV fraction 8% mol), ATCC13869/pDXW-8-*phaCAB*, ATCC13869/pDXW-8, WM001/pDXW-8-*phaCAB*,WM001/pDXW-8; **b** PHB and PHBV yield of *C. glutamicum* recombinants; **c** Intracellular acetyl-CoA and propionyl-CoA levels of ATCC13869, ATCC13869/pDXW-8-*phaCAB*, WM001, and WM001/pDXW-8-*phaCAB*. Each measurement was individually repeated three times

Since a huge propionyl-CoA pool was discovered in WM001, the PHA produced by WM001 recombinant could be PHBV. The intracellular PHA of WM001/pDXW-8, WM001/pDXW-8-*phaCAB*, ATCC13869/pDXW-8, and ATCC13869/pDXW-8-*phaCAB* were extracted and analyzed by gas chromatography (Fig. 4a). Standard PHBV with the 8% (mol/mol) 3HV fraction yielded two peaks at 8.06 and 9.07 min, and the former is much larger, suggesting that the 8.06 min peak is derived from 3HB while the other from 3HV. Both 8.05 and 9.06 min peaks were observed in the spectrum of PHA from WM001/pDXW-8-*phaCAB,* and the area of the 9.06 min peak is larger than 8.05 min peak indicating that PHBV synthesized by WM001/pDXW-8-*phaCAB* contains a high level of 3HV. In contrast, PHA samples from ATCC13869/pDXW-8-*phaCAB* showed a single 8.06 min peak and no 9.06 min peak (Fig. 4a), which was consistent with the previous study [52] and confirmed that ATCC13869/pDXW-8-*phaCAB* could not produce PHBV under the cultivation condition in this study. Two control samples showed no peaks, suggesting that PHB and PHBV were originated from *phaCAB* overexpression. The quantitative results showed that WM001 recombinant produced PHBV [28.7% (w/w), 58.1% (mol/mol) 3HV, 9.4 g/L] while the wild-type ATCC13869 recombinant produced PHB [20.0% (w/w), 6.7 g/L] (Fig. 4b). On the other hand, the 3HV and 3HB esters were confirmed by GC/MS (Additional file 1: Figure S3).

To confirm the intracellular propionyl-CoA pool in WM001, intracellular CoA esters of WM001 and ATCC13869 cells were extracted and quantified with LC–MS. In WM001, propionyl-CoA increased 16.9-fold (from 0.08 to 1.35 μM) and acetyl-CoA increased 1.3-fold (from 3.80 to 5.00 μM). Meanwhile, the ratio of propionyl-CoA to acetyl-CoA increased 12-fold from 0.02 to 0.25 (Fig. 4c). The transcriptome analysis and the LC–MS quantification results of propionyl-CoA confirmed the existence of a huge propionyl-CoA pool in WM001. Where does the excessive propionyl-CoA come from? According to previous publications, there are at least six sources for propionyl-CoA production. (I) The β-oxidation, using fatty acid as substrate, this pathway does not exist in *C. glutamicum* [22]; (II) the propionate degradation, the most common source of propionyl-CoA. *C. glutamicum* could not produce PHBV without propionate in ATCC13869 [22]. Our results of ATCC13869 producing PHB were consistent with this study (Fig. 4a). On the other aspect, extracellular propionate of WM001/pDXW-8-*phaCAB* and the control was determined before inoculation (4.23 and 4.23 g/L) and after fermentation (2.39 and 2.46 g/L). The propionate was probably brought in by corn steep liquor which is essential for biotin auxotroph *C. glutamicum*. More importantly, the 3HV in PHBV copolymer was way more than propionate consumed in the medium, needless to say, ATCC13869 recombinant produced PHB, not PHBV with the same medium; (III) the 2-ketobutyrate pathway, propionyl-CoA could be synthesized from 2-ketobutyrate by pyruvate dehydrogenase complex. But the conversion rate of 2-ketobutyrate to propionyl-CoA is ten times slower than that of pyruvate to acetyl-CoA [53]. As 2-ketobutyrate is one of the crucial intermediates in l-isoleucine synthesis, there might be excessive 2-ketobutyrate in WM001, and could be channeled towards propionyl-CoA synthesis; (IV) the (2R)-methylmalonyl-CoA pathway, in which succinyl-CoA could convert to propionyl-CoA through *sbm* and *ygfG* genes [54], but this coenzyme vitamin B_12_ dependent pathway does not exist in *C. glutamicum*; (V) the levulinyl-CoA pathway, which was identified in *C. necator*. The acetyl-CoA and propionyl-CoA could be converted from levulinate [55] and used for PHBV production in *C. necator* [56]. (VI) other pathways, in *Rhodococcus aetherivorans*, PHBV could be produced with toluene or acetate but the mechanism remains obscure [47]. Among these above mentioned six possible sources, we intend to believe the excessive propionyl-CoA in WM001 might comes from 2-ketobutyrate (Source III).

**Fig. 5:**
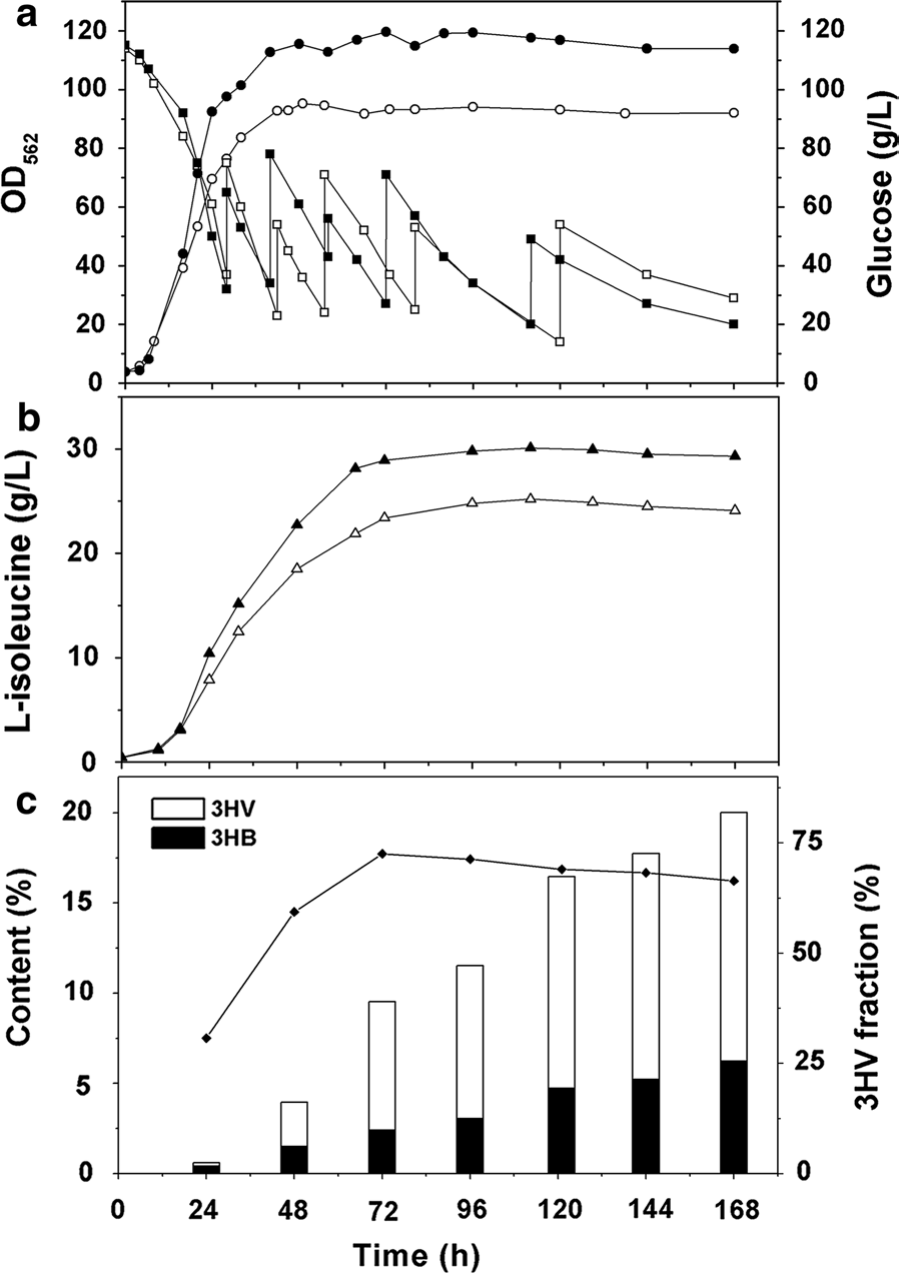
Fed-batch fermentation profiles of *C. glutamicum* WM001/pDXW-8 and WM001/pDXW-8-*phaCAB*. **a** OD_562_ of WM001/pDXW-8-*phaCAB* (solid circle) and WM001/pDXW-8 (open circle), residual glucose of WM001/pDXW-8-*phaCAB* (solid square) and WM001/pDXW-8 (open square); **b** l-isoleucine concentration produced by WM001/pDXW-8-*phaCAB* and WM001/pDXW-8; **c** PHBV content and 3HV fractions (solid diamond) of PHBV

In previous studies, PHB accumulation enhanced the synthesis of the co-products, as PHB consumed acetyl-CoA as the substrate, it could also enhance metabolites related to acetyl-CoA such as l-glutamate [23] and l-arginine [25]. In this study, we observed 8.9 and 10.6% increase of acetyl-CoA in WM001/pDXW-8-*phaCAB* and ATCC13869/pDXW-8-*phaCAB* than the control strains (Fig. 4c). The acetyl-CoA enhancement complemented with previous studies [23–25], suggesting that PHA production not only consumed acetyl-CoA substrate but also replenish the acetyl-CoA pool, that could explain the enhancement of these metabolites while PHA co-producing in the cells. On the other hand, the excessive acetyl-CoA was channeled towards the down-regulated TCA cycle of WM001 (Fig. 1), that might be the reason that WM001/pDXW-8-*phaCAB* exhibit better growth than the control (Figs. 3c and 5a).

### Co-production of l-isoleucine and PHBV in fed-batch fermentation

Fed-batch fermentation of WM001/pDXW-8-*phaCAB* was performed, using WM001/pDXW-8 as the control. During the fermentation, the pH was kept between 7.2 and 7.4 because the fermentation would stop before stationary phase if the pH were lower than 7.0 according to our experiences. Glucose consumption of the two recombinants was similar (Fig. 5a). After 24 h, l-isoleucine concentration rapidly increased, and PHBV started to accumulate (Fig. 5c). At 24 h, the 3HV mole fraction was only 31%, as l-isoleucine rapidly accumulated during 24 and 72 h (Fig. 5b), the 3HV fraction sharply raised to 59% (48 h) and 72.5% (72 h) (Fig. 5c). The 3HV fraction at 72 h is 280% higher than the previous highest 3HV fraction with glucose as the only carbon source (Table 1). During 72 and 168 h, the 3HV fraction decreased and reached 66.3% at the end of the fermentation (Fig. 5c). At 168 h, WM001/pDXW-8-*phaCAB* produced 20.0% PHBV with 66.2% (mol/mol) 3HV. As a result, WM001 recombinant produced PHBV with the 3HV fraction ranges from 31 to 72.5%. 3HV fraction increased sharply as the l-isoleucine rapidly accumulated, suggesting that the 3HV metabolism was coupled with l-isoleucine metabolism, which complemented our assumption that the excessive propionyl-CoA comes from 2-ketobutyrate.

As for l-isoleucine production, WM001/pDXW-8-*phaCAB* produced 29.8 g/L l-isoleucine, while the control produced 24.3 g/L at 96 h (Fig. 5b). This titer is quite close to the highest reported titer i.e. 32.3 g/L [57]. The yield on glucose increased 27% to 0.129 g/g glucose which is also close to the highest, i.e. 0.137 g/g [58], thus indicating that WM001/pDXW-8-*phaCAB* consumed glucose economically to produce both l-isoleucine and PHBV. Moreover, PHA accumulation depended on high C:N ratio, the l-isoleucine yield reached the maximum at 96 h, but PHBV yield reached the maximum until 168 h mostly because of the adequate nitrogen source at the beginning, when nitrogen source was depleted by l-isoleucine production, C:N ratio increased, and the supplied carbon source was used for PHBV production.

Our results showed that PHBV accumulation increased 44 and 23% l-isoleucine production in batch and fed-batch fermentation. The yield on glucose also increased 65 and 27%. With the high yield of both l-isoleucine and PHBV with the low-cost medium, *C. glutamicum* WM001 recombinant has a great industrial potential for PHBV and l-isoleucine co-production. With glucose as the only carbon source, WM001/pDXW-8-*phaCAB* could produced PHBV with a high 3HV fraction, which helps saving the cost of the addition of related precursors of PHBV (Table 1). Furthermore, the excessive propionyl-CoA could be channeled into other propionyl-CoA derivatives such as 1-propanol, 1-butanol, and higher alcohols. With the genome and transcriptome analyzed, *C. glutamicum* WM001 could act as a Gram-positive platform microorganism. As for the further enhancement of PHBV productivity, the fermentation parameters need to be optimized aiming at high-density culture, along with integrated omics analysis and fine-tuned metabolic flux, WM001 would become a promising PHBV and l-isoleucine microbial producer.

## Conclusions

According to the omics analysis, we preliminary revealed the mechanisms of l-isoleucine over-production in WM001. More importantly, the unexpected activated propionyl-CoA catabolism was detected and an assumed huge propionyl-CoA pool was discovered and confirmed. In WM001, propionyl-CoA increased 16.9-fold and the ratio of propionyl-CoA to acetyl-CoA increased 12.5-fold compared to the wild-type. After *phaCAB* cluster was introduced, the recombinant strain produced 15.0 g/L PHBV with 66.3% 3HV in fed-batch fermentation during which the maximum 3HV fraction reached 72.5%, which is 280% higher than the previous highest 3HV fraction with glucose. Thus, WM001 was characterized as an efficient l-isoleucine and PHBV co-producer and potential producers for propionyl-CoA derivatives.

## Additional file

**Additional file 1: Figure S1.** SEM analysis of WM001/pDXW-8 and WM001/pDXW-8-*phaCAB*. **Figure S2.** SDS-PAGE of WM001 recombinants: Lane 1, WM001/pDXW-8-*phaCAB*; Lane 2, WM001/pDXW-8-*phaAB*; Lane 3, WM001/pDXW-8-*phaA*; Lane 4, WM001/pDXW-8; Lane 5, marker. **Figure S3.** GC/MS analysis of PHA produced by WM001/pDXW-8-*phaCAB*. **Table S1.** Batch fermentation of WM001 recombinants after 72 h. **Table S2.** Differentially expressed genes between WM001 and ATCC13869.

## Abbreviations

PHA(s): polyhydroxyalkanoate(s)
PHB: poly(3-hydroxybutyrate)
PHBV: poly(3-hydroxybutyrate-co-3-hydroxyvalerate)
DCW: dry cell weight
TEM: transmission electron microscopy
SEM: scanning electron microscopy
