# Supplementary material for: Poly(3-hydroxybutyrate-co-3-hydroxyvalerate) co-produced with l-isoleucine in Corynebacterium glutamicum WM001
Source: Microb Cell Fact. 2018 Jun 15;17:93. doi: 10.1186/s12934-018-0942-7 (PMC6004086; doi:10.1186/s12934-018-0942-7)
Supplement: Supplementary file 1 — Additional file 1: Figure S1. SEM analysis of WM001/pDXW-8 and WM001/pDXW-8-phaCAB. Figure S2. SDS-PAGE of WM001 recombinants: Lane 1, WM001/pDXW-8-phaCAB; Lane 2, WM001/pDXW-8-phaAB; Lane 3, WM001/pDXW-8-phaA; Lane 4, WM001/pDXW-8; Lane 5, marker. Figure S3. GC/MS analysis of PHA produced by WM001/pDXW-8-phaCAB. Table S1. Batch fermentation of WM001 recombinants after 72 h. Table S2. Differentially expressed genes between WM001 and ATCC13869. [file 12934_2018_942_MOESM1_ESM.docx]

**Figure S1** SEM analysis of WM001/pDXW-8 and WM001/pDXW-8-*phaCAB*.


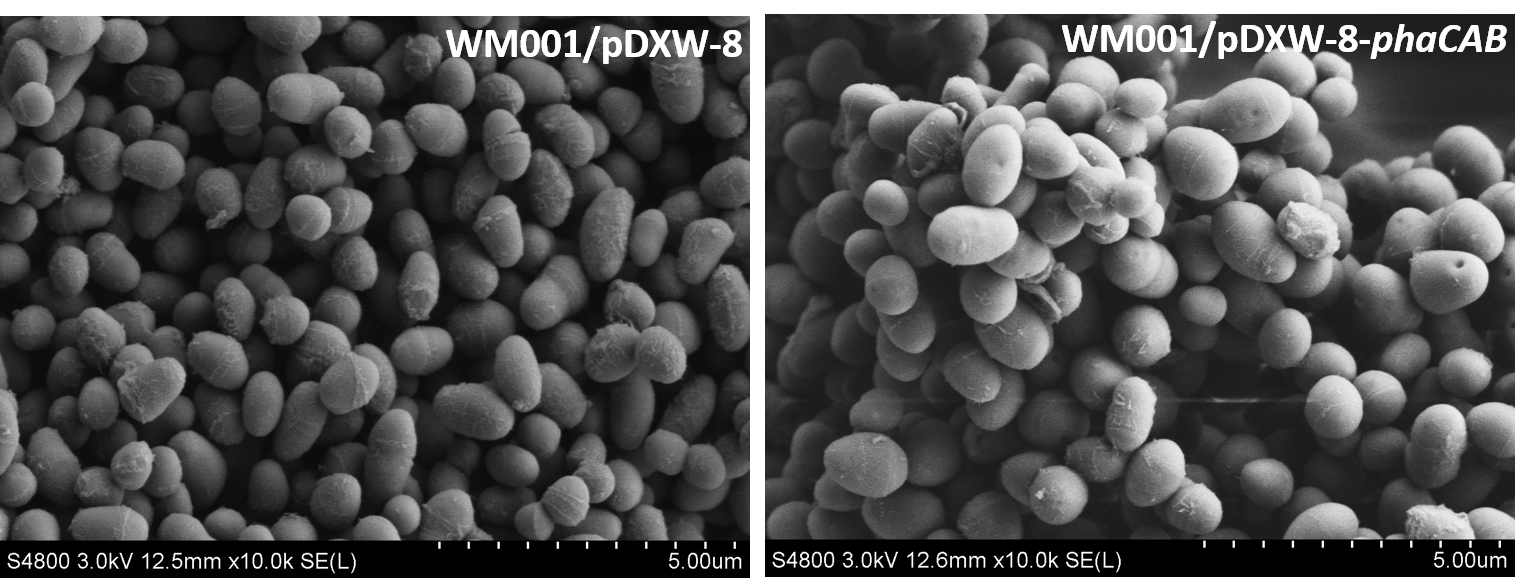


**Figure S2** SDS-PAGE of WM001 recombinants: Lane 1, WM001/pDXW-8-*phaCAB*; Lane 2, WM001/pDXW-8-*phaAB*; Lane 3, WM001/pDXW-8-*phaA*; Lane 4, WM001/pDXW-8; Lane 5, marker

**Figure S3 GC/MS analysis of PHA produced by WM001/pDXW-8-*phaCAB*.**

**Table S1 Batch fermentation of WM001 recombinants after 72 h.**

| Strains | WM001/pDXW-8 | WM001/pDXW-8-*phaA* | WM001/pDXW-8-*phaAB* | WM001/pDXW-8-*phaCAB* |
| --- | --- | --- | --- | --- |
| L-isoleucine (g/L) | 6.23±0.12 | 6.74±0.30 | 6.38±0.38 | 8.71±0.47 |

**Table S2 Differentially expressed genes between WM001 and ATCC13869**

| **Gene Name** | **log_2_Ratio** | **Genes Description** | **Genes length** | **Gene Location** |
| --- | --- | --- | --- | --- |
| **Propionyl-CoA metabolism** | |  |  |  |
| *prpB2* | 8.41↑ | methylisocitrate lyase | 924 | 674129-675052 |
| *prpC2* | 6.71↑ | 2-methylcitrate synthase ;citrate synthase | 1152 | 675081-676232 |
| *prpD2* | 5.83↑ | 2-methylcitrate dehydratase | 1515 | 672615-674129 |
| *cg2796* | 5.69↑ | 2-methylcitrate dehydratase | 1374 | 2656593-2657966 |
| *dtsR1* | 2.18↑ | propionyl-CoA carboxylase beta chain | 1632 | 731797-730166 |
| **TCA cycle** | |  |  |  |
| *sdhCD* | -2.39↓ | succinate dehydrogenase cytochrome b556 subunit | 774 | 392705-393478 |
| *icd* | -1.09↓ | isocitrate dehydrogenase | 2217 | 681517-679301 |
| *sucC* | 3.29↑ | succinyl-CoA synthetase beta subunit | 1209 | 2698260-2697052 |
| *sdhB* | -2.63↓ | succinate dehydrogenase iron-sulfur subunit | 750 | 395516-396265 |
| *acn* | -2.09↓ | aconitate hydratase 1 | 2832 | 1627746-1630577 |
| *mdh* | -1.06↓ | malate dehydrogenase | 987 | 2494924-2493938 |
| *sucD* | 2.53↑ | succinyl-CoA synthetase alpha subunit | 885 | 2697030-2696146 |
| *sdhA* | -2.53↓ | succinate dehydrogenase flavoprotein subunit | 2022 | 393495-395516 |
| *fumC* | -1.43↓ | fumarate hydratase, class II | 1410 | 1065086-1063677 |
| *gltD* | -3.25↓ | glutamate synthase (NADPH/NADH) small chain | 1521 | 199773-201293 |
| *gltB* | -2.92↓ | glutamate synthase (NADPH/NADH) large chain | 4533 | 195241-199773 |
| *glnA* | -1.33↓ | glutamine synthetase | 1434 | 2320501-2321934 |
| **Oxidative phosphorylation** | |  |  |  |
| *cydB* | 1.93↑ | cytochrome d ubiquinol oxidase subunit II | 1002 | 1203556-1202555 |
| *cydA* | 1.43↑ | cytochrome d ubiquinol oxidase subunit I | 1542 | 1205100-1203559 |
| *sdhB* | -2.63↓ | succinate dehydrogenase iron-sulfur subunit | 750 | 395516-396265 |
| *sdhA* | -2.53↓ | succinate dehydrogenase flavoprotein subunit | 2022 | 393495-395516 |
| *sdhCD* | -2.39↓ | succinate dehydrogenase cytochrome b556 subunit | 774 | 392705-393478 |
| *ctaC* | -2.25↓ | cytochrome c oxidase subunit II | 1080 | 2299669-2298590 |
| *qcrB* | -1.91↓ | ubiquinol-cytochrome c reductase cytochrome b subunit | 1620 | 2294760-2293141 |
|  |  |  |  |  |
| *ctaE* | -1.48↓ | cytochrome c oxidase subunit III | 618 | 2297559-2296942 |
| *qcrC* | -1.48↓ | ubiquinol-cytochrome c reductase cytochrome c subunit | 888 | 2296867-2295980 |
| *qcrA1* | -1.45↓ | ubiquinol-cytochrome c reductase iron-sulfur subunit | 1227 | 2295983-2294757 |
| *ndh* | -1.24↓ | NADH dehydrogenase | 1404 | 1546021-1544618 |
| *ctaD* | -1.11↓ | cytochrome c oxidase subunit I | 1755 | 2644488-2642734 |
| **Glycolysis pathway** | |  |  |  |
| *bglY* | 1.76↑ | 6-phospho-beta-glucosidase | 363 | 3003367-3003005 |
| *ppgK* | -1.49↓ | polyphosphate glucokinase | 753 | 1980681-1981433 |
| *cg2455* | -1.16↓ | probable phosphoglycerate mutase | 1149 | 2344230-2343082 |
| **Pyruvate metabolism** | |  |  |  |
| *cg3096* | 4.37↑ | aldehyde dehydrogenase (NAD+) | 1521 | 2955004-2953484 |
| *aceB* | 2.86↑ | malate synthase | 2220 | 2441817-2439598 |
| *cg2266* | 1.41↑ | acylphosphatase | 285 | 2153423-2153139 |
| *aceE* | 1.22↑ | pyruvate dehydrogenase E1 component | 2769 | 2351652-2354420 |
| *pck* | 1.01↑ | phosphoenolpyruvate carboxykinase (GTP) | 1833 | 3027197-3025365 |
| *mqo* | -1.43↓ | malate dehydrogenase (quinone) | 1503 | 2085413-2083911 |
| *mdh* | -1.06↓ | malate dehydrogenase | 987 | 2494924-2493938 |
| **Amino acids biosynthesis** | |  |  |  |
| *ilvN* | 2.85↑ | acetolactate synthase small subunit | 519 | 1341494-1342012 |
| *ilvB* | 2.45↑ | acetolactate synthase large subunit | 1881 | 1339600-1341480 |
| *leuA* | 1.46↑ | 2-isopropylmalate synthase | 1746 | 267897-266152 |
| *leuD* | -2.46↓ | 3-isopropylmalate/(R)-2-methylmalate dehydratase small subunit | 594 | 1383369-1383962 |
| *leuC* | -2.13↓ | 3-isopropylmalate/(R)-2-methylmalate dehydratase large subunit | 1446 | 1381907-1383352 |
| *ilvE* | -1.34↓ | branched-chain amino acid aminotransferase | 1140 | 2308723-2307584 |
| *thrB* | 2.72↑ | homoserine kinase | 930 | 1245323-1246252 |
| *hom* | 2.26↑ | homoserine dehydrogenase | 1338 | 1243975-1245312 |
| *soxA* | -2.65↓ | sarcosine oxidase ;  N-methyl-L-tryptophan oxidase | 408 | 1673144-1672737 |
| *serC* | -2.27↓ | phosphoserine aminotransferase | 1131 | 878583-877453 |
| *glyA* | -1.45↓ | glycine hydroxymethyltransferase | 1305 | 1052094-1053398 |
| *panB* | -1.17↓ | 3-methyl-2-oxobutanoate  hydroxymethyltransferase | 816 | 128005-127190 |
| *cg0931* | 1.35↑ | N-succinyldiaminopimelate aminotransferase | 1164 | 863063-864226 |
| *metY* | 1.08↑ | O-acetylhomoserine (thiol)-lyase | 1314 | 669231-667918 |
| *cg0961* | 1.86↑ | homoserine O-acetyltransferase | 1050 | 898113-897064 |
| *cg2337* | -1.39↓ | 5-methyltetrahydropteroyltriglutamate--homocysteine methyltransferase | 1218 | 2218252-2217035 |
| *metE* | -1.29↓ | 5-methyltetrahydropteroyltriglutamate--homocysteine methyltransferase | 2238 | 1192090-1189853 |
| *cg1759* | 1.51↑ | serine O-acetyltransferase; ring-1,2-  phenylacetyl-CoA epoxidase subunit PaaD | 414 | 1649089-1648676 |
| *cg3390* | 4.15↑ | 4-hydroxyphenylpyruvate dioxygenase | 882 | 3236545-3237426 |
